# Highly active antiretroviral therapy is necessary but not sufficient. A systematic review and meta-analysis of mortality incidence rates and predictors among HIV-infected adults receiving treatment in Ethiopia, a surrogate study for resource-poor settings

**DOI:** 10.1186/s12889-024-19268-1

**Published:** 2024-06-28

**Authors:** Beshada Zerfu Woldegeorgis, Yordanos Sisay Asgedom, Aklilu Habte, Gizachew Ambaw Kassie, Abebe Sorsa Badacho

**Affiliations:** 1 Department of Internal Medicine, College of Health Sciences and Medicine, Wolaita Sodo University, Wolaita Sodo, Ethiopia; 2 Department of Epidemiology, College of Health Sciences and Medicine, Wolaita Sodo University, Wolaita Sodo, Ethiopia; 3 School of Public Health, College of Medicine and Health Sciences, Wachemo University, Hosanna, Ethiopia; 4 School of Public Health, College of Health Sciences and Medicine, Wolaita Sodo University, Wolaita Sodo, Ethiopia

**Keywords:** Incidence, Mortality, Predictors, Human immune deficiency virus, Antiretroviral therapy, Meta-analysis

## Abstract

**Background:**

Owing to the introduction of highly active antiretroviral therapy (HAART), the trajectory of mortality and morbidity associated with human immunodeficiency virus (HIV) infection has significantly decreased in developed countries. However, this remains a formidable public health challenge for people living with HIV in resource-poor settings. This study was undertaken to determine the pooled person-time incidence rate of mortality, analyze the trend, and identify predictors of survival among HIV-infected adults receiving HAART.

**Methods:**

Quantitative studies were searched in PubMed, Embase, Scopus, Google Scholar, African Journals Online, and Web of Science. The Joana Briggs Institute critical appraisal tool was used to assess the quality of the included articles. The data were analyzed using the random-effects Dersimonian-Laird model.

**Results:**

Data abstracted from 35 articles involving 39,988 subjects were analyzed. The pooled person-time incidence rate of mortality (all-cause) was 4.25 ([95% uncertainty interval (UI), 3.65 to 4.85]) per 100 person-years of observations. Predictors of mortality were patients aged ≥ 45 years (hazard ratio (HR), 1.70 [95% UI,1.10 to 2.63]), being female (HR, 0.82 [95% UI, 0.70 to 0.96]), history of substance use (HR, 3.10 [95% UI, 1.31 to 7.32]), HIV positive status non disclosure (HR, 3.10 [95% UI,1.31 to 7.32]), cluster of differentiation 4 + T cell - count < 200 cells/mm3 (HR, 3.23 [95% UI, [2.29 to 4.75]), anemia (HR, 2.63 [95% UI, 1.32 to 5.22]), World Health Organisation classified HIV clinical stages III and IV (HR, 3.02 [95% UI, 2.29 to 3.99]), undernutrition (HR, 2.24 [95% UI, 1.61 to 3.12]), opportunistic infections (HR, 1.89 [95% UI, 1.23 to 2.91]), tuberculosis coinfection (HR, 3.34 [95% UI, 2.33 to 4.81]),bedridden or ambulatory (HR,3.30 [95% UI, 2.29 to 4.75]), poor treatment adherence (HR, 3.37 [95% UI,1.83 to 6.22]), and antiretroviral drug toxicity (HR, 2.60 [95% UI, 1.82 to 3.71]).

**Conclusion:**

Despite the early introduction of HAART in Ethiopia, since 2003, the mortality rate has remained high. Therefore, guideline-directed intervention of identified risk factors should be in place to improve overall prognosis and increase quality-adjusted life years.

**Supplementary Information:**

The online version contains supplementary material available at 10.1186/s12889-024-19268-1.

## Introduction

Globally, despite an overall decline in the reported prevalence, the human immune deficiency virus (HIV) continues to afflict more than 39 million people (37.5 million were 15 years of age or older) in 2022, and an estimated 1.3 million incident cases (1.2 million were 15 years of age or older) and 630,000 deaths from HIV-related illnesses were registered in the same year [1].

Africa is home to an estimated 25.6 million HIV-infected people and 60% of global acquired immunodeficiency syndrome (AIDS) deaths [2]. Due to the introduction and scaling-up of HAART, HIV-related mortality has steadily declined over the past two decades in developed countries; however, the problem remains important in low-resource settings, including Ethiopia [2, 3]. In sub-Saharan Africa (SSA), which is home to an estimated 67% of the global HIV-infected population and 76% of global AIDS deaths, the proportion of early mortality among adults accessing HAART was very high; between 6 and 26% of patients died [4], which ascertains that treatment of HIV is still a challenge in resource-poor settings (5.55 deaths per 100 person-years of observation (PYO) compared to resource-rich settings (2 deaths per 100 PYO) [5].

In Ethiopia, an estimated 603,537 people were living with HIV (570,511 were 15 years of age or older), and annual AIDS deaths were estimated at 9,984 (approximately 86% were 15 years of age or older), according to the Ethiopian Public Health Institute HIV estimates and projections for the year 2023 [6]. With the introduction of HAART in resource-limited settings in the early 2000s, Ethiopia was among the first African countries to introduce HAART in 2003 in selected health facilities. With the free HAART program in early 2005, a significant number of deaths have been averted due to the concerted efforts of the government and its partners, and HAART coverage for 15 years of age or older has reached 82% in 2022, and the country is striving to attain the 95-95-95 global goal by 2030 [7].

The provision of HAART is necessary, but not sufficient to increase survival among patients receiving treatment, and evidence suggests that timely diagnosis, assessment of eligibility, and provision of treatment free of charge are associated with a lower risk of mortality [5]. Furthermore, evidence from various studies in low settings suggests that advanced HIV/AIDS at presentation [4, 5, 8–11], low quality of health service care [4], undernutrition [8, 9, 12–15], anemia [8, 9, 12, 16], sex [8, 9, 13, 17, 18], tuberculosis (TB)-HIV co-infection at enrollment [19, 20], and poor HAART adherence [21, 22] were predictors of mortality among adults receiving HAART.

Although there has been no nationally representative summary data, estimates from individual studies conducted in health facilities providing chronic HIV care and treatment services in Ethiopia revealed a person-time incidence rate of mortality between 0.28 deaths per 100 persons per year [23] in Suhul Hospital in the Tigray region and 22.9 deaths per 100 persons per year in DebreMarkos Referral Hospital in the Amhara region [24]. To better understand the success of the HIV program in Ethiopia and inform policymakers, we aimed answers to the following questions: (1) What is the pooled person-time incidence rate of mortality among HIV-infected adult patients initiating HAART in Ethiopia? (2) What are the predictors of mortality among adult HIV-infected patients initiating HAART in Ethiopia? (3) What is the trend of death over time in adult patients initiating HAART in Ethiopia?

## Methods and materials

### Study protocol registration and reporting

A full study protocol, written based on the Preferred reporting items for systematic review and meta-analysis protocols 2015 [25], submitted to the Prospective Register of Systematic Reviews and registered with registration number CRD42023481380. We reported the systematic review and meta-analysis results using the Preferred Reporting Items for Systematic Reviews and Meta-Analyses (PRISMA) 2020 checklist [26] (Additional file 1).

### Eligibility criteria

#### Population/type of participants

Persons living with HIV (PLHIV) aged 15 or older and initiating HAART in Ethiopia were considered.

#### Condition/domain

Articles that described the outcome of the interest based on PLHIV survival and predictors of mortality after initiating HAART were considered.

#### Context/settings

Follow up studies (retrospective/prospective) conducted in Ethiopia and published in the English language from inception to August 31, 2023, were included. Otherwise, articles without full-text access; articles that did not contain required information on the outcomes of interest; studies published in non-open access journals; findings from personal opinions; articles reporting outside the scope of the outcome of interest; qualitative study design; case reports; case series; letters to editors; and unpublished data were excluded.

### Information sources and search strategy

A double-blinded search was carried out by two authors (BZW and YSA) from March 1, 2023, to August 31, 2023, in the Excerpta Medica database, PubMed, Web of Science, African Journals Online, Google Scholar, and Scopus. Furthermore, the reference lists of final articles included in the quantitative synthesis were scanned to ensure literature saturation. Literature search strategies were developed using medical subject headings and text words related to the outcomes of interest. The search terms employed include: “ mortality”, “death”, “survival”, “HIV/AIDS”, “Human immune deficiency virus”, “acquired immune deficiency syndrome”, “ART”, “antiretroviral therapy”, “HAART”, “highly active antiretroviral therapy”, “prevalence,” “proportion”, “incidence”, “associated factors”, “predictors”, “determinants”, “adults”, adolescents, and “Ethiopia” (S1 Table).

### Study selection procedures

Articles were exported to the reference management software, EndNote X7, where duplicate studies were then eliminated. Two authors (AH and GAK) independently screened the titles and abstracts. The screened articles were then subjected to a full article review by two independent authors (AG and AK). Pre-specified criteria for inclusion in the review were followed to determine which records were relevant and should be included. Where more information was required to answer queries regarding eligibility, the remaining authors were involved. Disagreements about whether a study should be included were resolved by discussion. Moreover, the reasons for excluding the articles were recorded at each step.

### Data extraction

Two authors (ASB and BZW), working independently, excerpted the relevant data from the studies using a standardized Microsoft Excel spreadsheet. For data extraction, Joana Briggs Institute data collection formats suitable for meta-analysis were employed [27]. The data extraction format captured data on the following main components: information about data extraction from reports (name of data extractors, date of data extraction, and study identification number), study authors, year of publication of the article, study methods (study design, statistical analysis), study settings (regions, and specific areas from which study participants recruited), population characteristics (sex, age), information related to the pre-specified outcome domain, measurement tool or instrument, and information related to the results for each study included in the quantitative analysis (number of participants included in the analysis, and the non-response rate). In the case of disagreements between the two data extractors, a third author (AH) was involved in adjudicating unresolved disagreements through discussion and re-checking of the original articles.

### Methodological quality assessment

Two authors (YSA and GAK) evaluated the original studies using the Joanna Briggs Institute critical appraisal checklist designed for cohort studies which included 11 constructs. The response options were labeled as ‘yes’, 'no', and 'unclear question'. The total score was computed by counting the number of 'yes' answers in each row. Articles with critical appraisal scores of 7 and above were included in the systematic review and meta-analysis (S2 Table).

### Outcome and effect measures

The primary outcome of interest was the person-time incidence rate of mortality. The pooled incidence density was computed as the number of deaths divided by the total number of years of observation multiplied by 100. The secondary outcome was predictors of mortality, and the hazard ratio was the summary effect measure employed. We categorized the predictor variables as follows: residence (rural vs. urban), age (< 45 vs. ≥ 45), sex (male vs.female), substance use (yes vs.no), HIV-positive status disclosure (yes vs. no), HAART adherence (poor vs. good and fair), cotrimoxazole preventive therapy (yes vs. no), tuberculosis preventive therapy (yes vs. no), hemoglobin (< 10 g/dl vs. ≥ 10 g/dl), opportunistic infections (OIs) (yes vs. no), body mass index (BMI) (< 18.5 vs. ≥ 18.5), the World Health Organisation (WHO) classified HIV clinical stages (III and IV vs.I and III), the cluster of differentiation(CD)4 + T lymphocyte count (< 200 vs. ≥ 200), TB-HIV co-infection (yes vs. no), functional status (working vs. ambulatory/bedridden), and antiretroviral drug (ARV) toxicity (yes vs.no).

### Data synthesis

Extracted data were imported from Microsoft Excel 2010 into Stata 16 MP version for analysis. The presence and extent of variability among studies (inconsistency or heterogeneity) were evaluated graphically (present when the uncertainty interval for the results of individual studies generally depicted in forest plots using the horizontal lines have poor overlap) and more formally, using statistical methods (the Cochrane chi-squared test, included in the forest plots, the threshold for statistical significance was set at *P* ≤ 0.1; Higgins and Thompson’s I^2^ statistics: 0% to 40%: may not be important; 30% to 60% may represent moderate heterogeneity; 50% to 90%: may represent substantial heterogeneity; 75% to 100%: considerable heterogeneity) [28]. We employed the random-effect meta-analysis model to estimate Der Simonian and Laird’s pooled effect, as considerable statistical heterogeneity was observed (Higgins and Thompson’s I^2^ statistics was ≥ 50% and *P*.value was ≤ 0.1). Subgroup analyses (based on sample size, and HAART eligibility as covariates), meta-regression (based on year of publication, and sample size as covariates), and sensitivity analyses were performed. To evaluate the presence of small study effects, publication bias was explored through statistical methods (Egger test: significant at *P* ≤ 0.05) and funnel plots [29]. Variables with *P* ≤ 0.05 were deemed statistically significant predictors of mortality, and the strength of the association was presented by HR with a corresponding 95% uncertainty interval (UI).

## Results

### Search and study selection

**Fig. 1:**
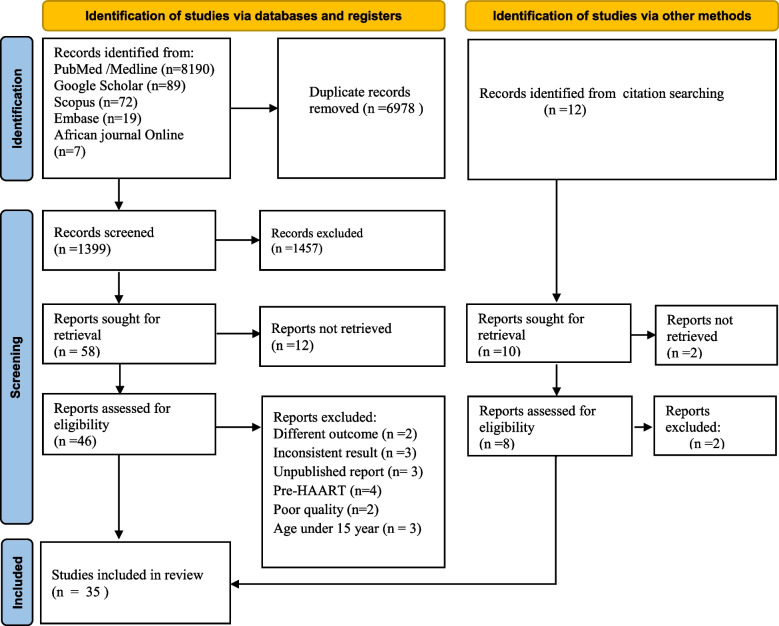
Thepreferred reporting items for systematic reviews and meta-analyses flow diagram

The database search identified 8377 articles. After 6978 duplicate records were removed, the remaining 1399 were screened based on their title and abstracts, with 1457 being removed as unrelated to the study domain. Fourty-six full-text articles were evaluated against eligibility criteria, and 17 of them were removed (different outcome, *n* = 2, inconsistent results, *n* = 3, unpublished reports, *n* = 3, pre-HAART, *n* = 4, poor quality, *n* = 2, and age < 15 years old, *n* = 3). Furthermore, through citation searching, six articles were retrieved. Finally, 35 articles were eligible for quantitative analysis (Fig. 1).

### Study characteristics

A total of 35 eligible studies [30–64], with 39,988 participants were included. The study sample size ranged from 272 [34] to 11,013 [60] individuals. An estimated 61% (*n* = 24,316) of participants were females. All epidemiological studies were cohort [30–64]. The participants contributed 91,866.861 PYO, and 4,050 deaths were recorded. Mean or median survival time was reported in 13 of the 35 studies [31, 33, 34, 40, 41, 43, 46, 47, 53, 55, 58, 59, 64]. There were two subnational studies as part of the Advanced Clinical Monitoring for HIV/AIDS in patients with HIV infection [52, 60]. Six of the studies were conducted in the Amhara region [30, 42, 44, 51, 54, 59], four in the Oromia region [32–34, 55]; three in Addis Ababa city administration [38, 43, 53]; 12 in the Southern Nations, Nationalities, and Peoples' Region (SNNPR) [31, 35, 36, 39–41, 45, 46, 57, 62–64], three in Tigray [37, 49, 50], three in Harari region [47, 48, 61], one each in Afar [56] and Somalia [58] regions. The overall proportion of mortality was 10.13%, and estimates from individual studies suggest the cumulative incidence of mortality ranged from 4.43% [33] to 29.68% [30]. About 69% (*n* = 24) of the studies were published after the implementation of the WHO’s universal test and treatment strategy [30, 33, 35, 37–44, 47, 48, 51, 52, 54–57, 59, 60, 62–64] (Table 1).

**Table 1:** Metadata of primary studies included in the systematic review and meta-analysis

**SN**	**Authors (reference)**	**Year**	**Region**	**Study design**	**Sample size**	**Sex**		**Follow-up period**	**Deaths**	**Person-years at risk**	**Mortality rates per 100 PYO**	**Score**
						**Male**	**Female**					
1	Gebremichael [30]	2020	Amhara	Retrospective patients clinical record review	647	224	423	2012 - 2017	192	2,911.5	6.6	10
2	Tsegaye and Worku [31]	2011	SNNPR	Retrospective patients clinical record review	5,664	2,477	3,187	2005-2009	473	6,352	7.45	10
3	Hambisa et al. [32]	2013	Oromia	Retrospective patients clinical record review	416	174	242	2005-2012	30	1,587	1.89	11
4	Abebe et al. [33]	2016	Oromia	Retrospective patients clinical record review	384	169	215	2010-2011	17	792.4	2.1	10
5	Alemu and Sebastián [34]	2010	Oromia	Retrospective patients clinical record review	272	117	155	2006-2008	28	409.9	6.83	11
6	Hailemariam et al. [35]	2016	SNNPR	Retrospective patients clinical record review	2178	995	1,183	2005-2013	196	6,619	2.96	11
7	Girum et al. [36]	2020	SNNPR	Retrospective patients clinical record review	500	164	336	2012-2019	48	1,632.6	2.94	11
8	Belay et al. [37]	2017	Tigray	Retrospective patients clinical record review	638	199	439	2010-2015	48	2,105.60	2.28	10
9	Tesfaye et al. [38]	2021	Addis Ababa	Retrospective patients clinical record review	432	178	254	2014-2019	91	1,025.17	8.88	10
10	Kebede et al. [39]	2020	SNNPR	Retrospective patients clinical record review	455	190	265	2006-2010	34	886.5	3.84	9
11	Wondimu et al. [40]	2020	SNNPR	Retrospective patients clinical record review	364	153	211	2007-2017	83	0.42	1976.2	10
12	Barata et al. [41]	2023	SNNPR	Retrospective patients clinical record review	441	177	264	2015-2020	53	5.62	943.2	10
13	Teshale et al. [42]	2021	Amhara	Retrospective patients clinical record review	475	198	277	2015-2019	45	846.271	5.32	10
14	Tesfayohannes et al. [43]	2022	Addis Ababa	Retrospective patients clinical record review	613	284	329	2014-2019	55	1,693	3.25	10
15	Ahunie et al. [44]	2017	Amhara	Retrospective patients clinical record review	698	236	453	2005-2014	35	1,801.6	1.25	10
16	Mulissa et al. [45]	2010	SNNPR	Retrospective patients clinical record review	1428	713	715	2003-2008	220	2,422.4	9.08	11
17	Setegn et al. [46]	2015	SNNPR	Retrospective patients clinical record review	2036	904	1132	2007-2012	120	5,912	2.03	10
18	Birhanu et al. [47]	2021	Harari	Retrospective patients clinical record review	610	253	356	2013-2018	67	1,410.7	4.75	10
19	Eticha and Gemeda [48]	2018	Harari	Retrospective patients clinical record review	513	212	301	2005-2015	61	2,123.9	2.87	10
20	Biadgilign et al. [49]	2019	Tigray	Retrospective patients clinical record review	295	135	160	2010-2014	37	1,3214.28	0.28	10
21	Tadesse et al. [50]	2014	Tigray	Retrospective patients clinical record review	520	225	295	2006 -2011	46	1,400	3.29	10
22	Workie et al. [51]	2021	Amhara	Retrospective patients clinical record review	542	257	285	2018	84	1,245	6.75	10
23	Fekade et al. [52]	2017	Subnational	Retrospective patients clinical record review	976	382	594	2009-2013	101	1,825	5.53	10
24	Mengesha et al. [53]	2014	Addis Ababa	Retrospective patients clinical record review	416	185	231	2008-2012	37	973.68	3.8	10
25	Birhanu et al. [54]	2021	Amhara	Retrospective patients clinical record review	458	193	265	2010-2018	55	1,406.8	3.91	11
26	Seyoum et al. [55]	2017	Oromia	Retrospective patients clinical record review	456	144	312	2006-2010	66	1,245	5.3	10
27	Salih et al. [56]	2023	Afar	Retrospective patients clinical record review	702	293	409	2010-2015	82	1,411	5.81	11
28	Yohannes et al. [57]	2019	SNNPR	Retrospective patients clinical record review	507	195	312	2013-2017	26	1,169.39	2.22	11
29	Damtew et al. [58]	2015	Somali	Retrospective patients clinical record review	784	326	458	2007-2011	87	1,608	5.41	11
30	Nigussie et al. [59]	2020	Amhara	Retrospective patients clinical record review	447	148	299	2013-2018	54	1,291	4.18	10
31	Getaneh et al. [60]	2022	Subnational	Retrospective patients clinical record review	11013	3861	7152	2007-2019	1134	21,638	5.28	10
32	Digaffe et al. [61]	2014	Harari	Retrospective patients clinical record review	655	217	438	2005-2008	74	1,913	3.87	9
33	Abuto et al. [62]	2021	SNNPR	Retrospective patients clinical record review	467	204	263	2013-2018	59	1,412.7	4.18	10
34	Tachbele and Ameni [63]	2016	SNNPR	Retrospective patients clinical record review	350	129	186	2010-2015	35	1,995	1.75	10
35	Sapa et al. [64]	2016	SNNPR	Retrospective patients clinical record review	1391	761	630	2010-2014	128	3,648	3.51	10

*Abbreviations*: *SNNPR* the Southern Nations Nationalities Peoples region, *PYO* Person years of observation

#### Mortality

**Fig. 2:**
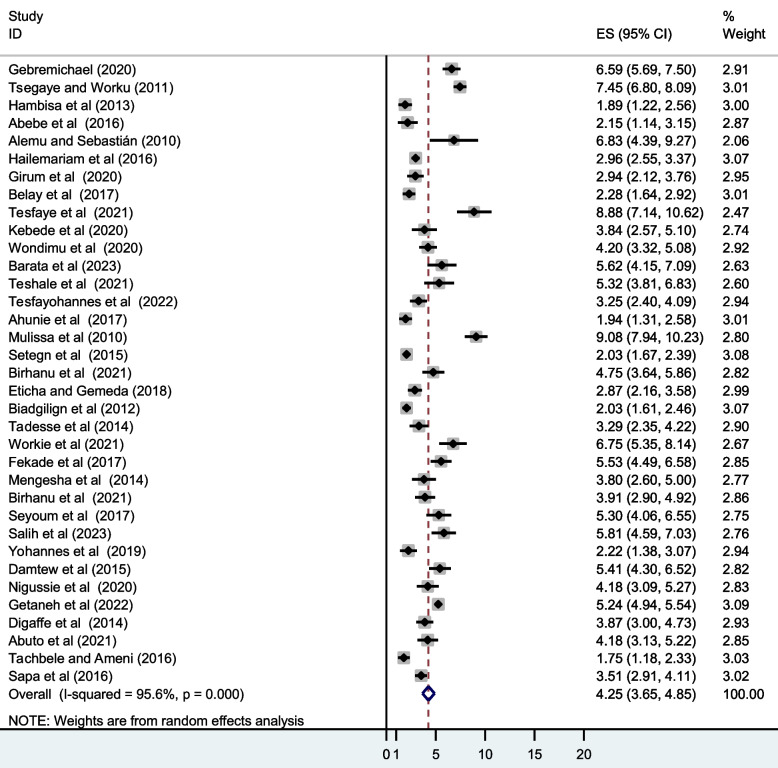
The pooled incidence density proportion of mortality among adult patients initiating highly active antiretroviral therapy in Ethiopia, 2023

The pooled person-time incidence rate of mortality among adult patients initiating HAART in Ethiopia was 4.25 deaths ([95% UI, 3.65,4.85]; I^2^ = 95.6%) per 100 PYO (Fig. 2).

#### Subgroup (subset) meta-analysis

**Fig. 3:**
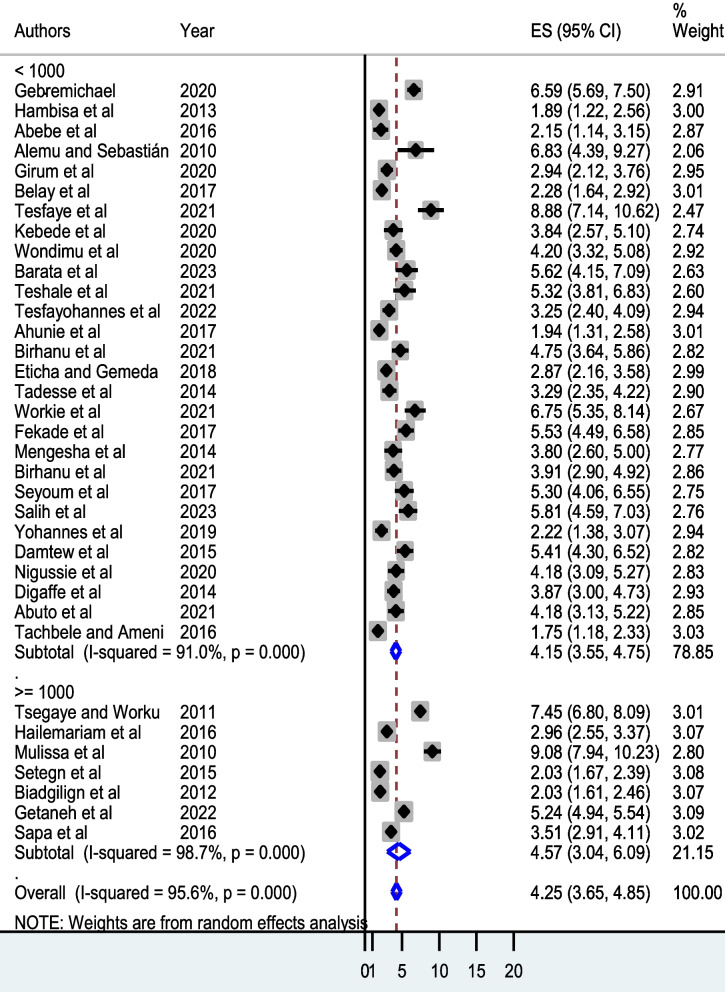
Subgroup meta-analysis by number of study participants among adult patients initiating highly active antiretroviral therapy in Ethiopia, 2023

**Fig. 4:**
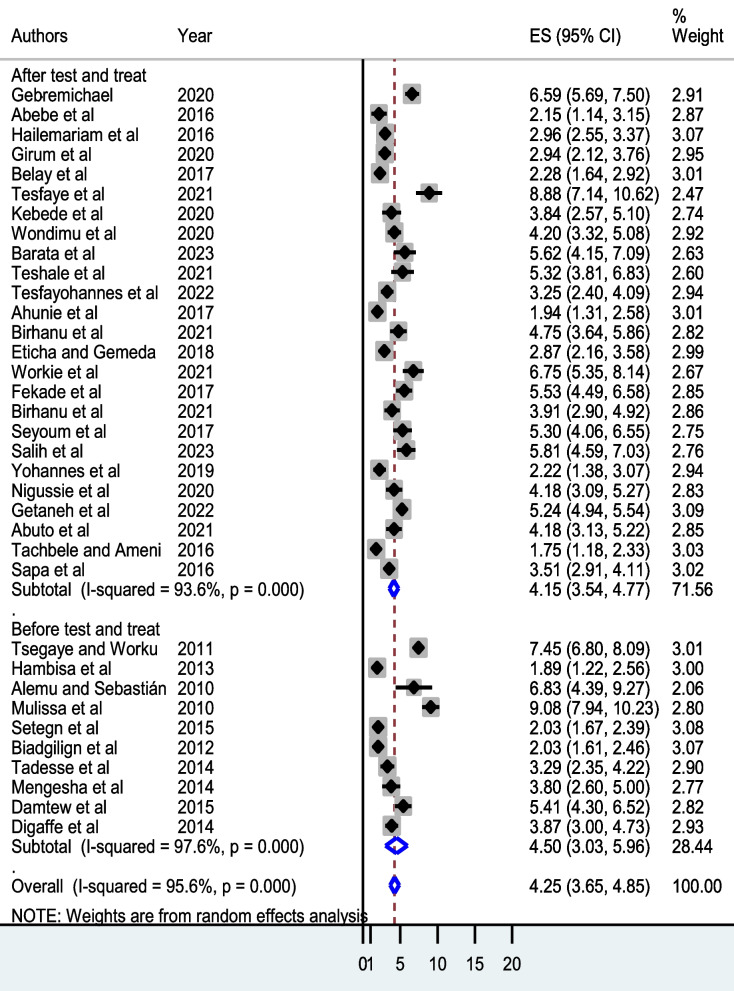
Subgroup meta-analysis by eligibility among adult patients initiating highly active antiretroviral therapy in Ethiopia, 2023

To identify the source of statistical heterogeneity, we undertook a subgroup random-effect meta-analysis for subsets of sample size partitioned into < 1000 and ≥ 1000 participants and HAART eligibility split into before and after the WHO’s universal test and treatment policy. The pooled person-time incidence rate of mortality was 4.57 deaths per 100 PYO (Fig. 3). Moreover, the pooled person-time incidence rate of mortality before the implementation of the WHO’s universal test and treatment policy was higher (4.50 deaths per 100 PYO) (Fig. 4).

#### Meta-regression

We further performed meta-regression analyses to explore the cause of heterogeneity, using the sample size and year of publication as covariates at 5% statistical significance. As illustrated in Table 2, these covariates were not found to be the cause of statistical heterogeneity.

**Table 2:** Meta‐regression analysis of factors affecting study heterogeneity

**Covariates**	**Coefficient**	**Standard error**	**t**	***P* >|t|**	**95% uncertainty interval**	
Sampe size	.0001753	.0001675	1.05	0.303	-.0001659	.0005165
Year of publication	.0281266	.0921374	0.31	0.762	-.1595511	.2158044

#### Sensitivity meta-analysis

**Fig. 5:**
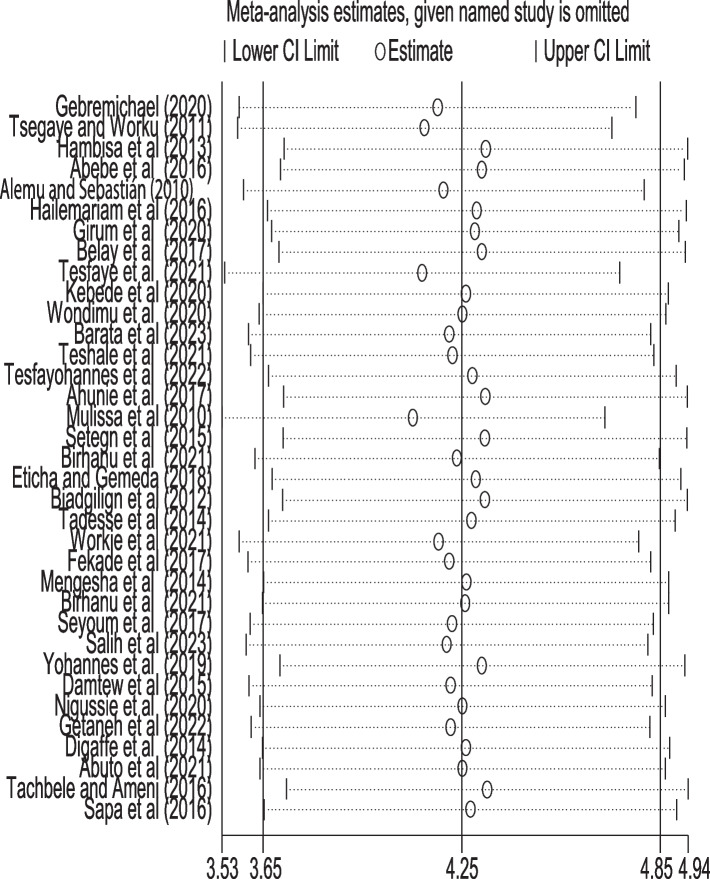
Illustration of sensitivity meta-analysis for adult patients initiating highly active antiretroviral therapy in Ethiopia, 2023

A leave-out-one sensitivity analysis was conducted to assess the impact of each study on the pooled incidence density of mortality while gradually excluding each study. The results showed that the combined effects did not change significantly as a result of the excluded study (Fig. 5).

#### Publication bias

**Fig. 6:**
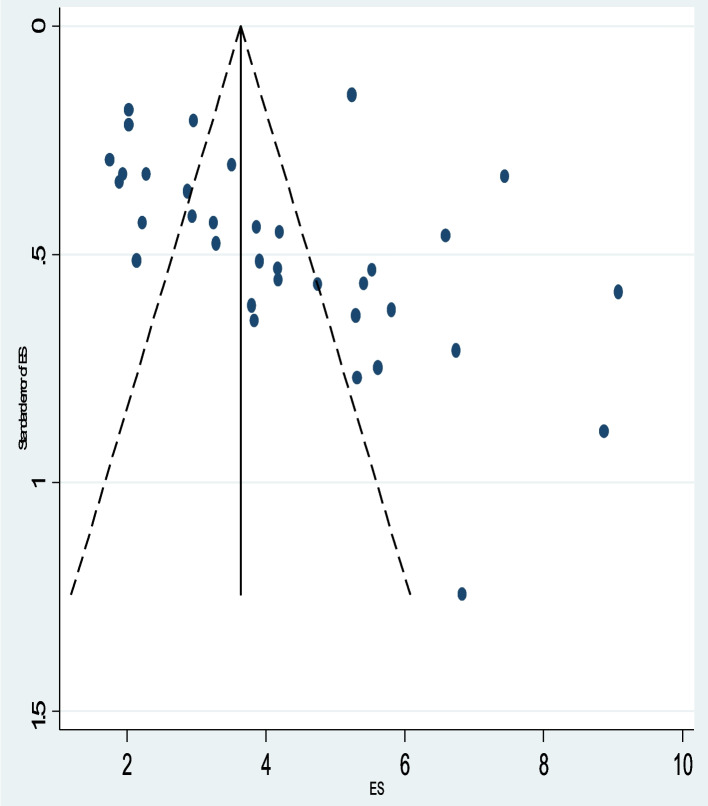
Funnel plots of publication biases. The x-axis shows the effect size, and the standard errors of the effect sizes were plotted on the y-axis. The dashed lines represent the 95% confidence interval. The dots show the distribution of individual studies. Studies with smaller sample sizes are scattered at the bottom of the funnel, and vice versa

To determine whether there is a possibility of publication bias or small-study effects, we looked at the distribution of studies about the summary effect sizes graphically using funnel plots. Thus, on inspection, the funnel plot showed there is no prominent asymmetrical distribution (Fig. 6).

Furthermore, the formal Egger linear regression test was not statistically significant (*P* = 0.080) corroborating the absence of evidence of small study effects (Table 3).

**Table 3:** Egger’s test for small study effects

**Standard effect**	**Coefficient**	**Standard error**	**t**	***P* >|t|**	**95% uncertainty interval**	
Slope	.9191311	.1660839	5.53	0.000	.5812309	1.257031
Bias	.8245211	.4564561	1.81	**0.080**	-.1041457	1.753188

#### Trends in estimated death rates

**Fig. 7:**
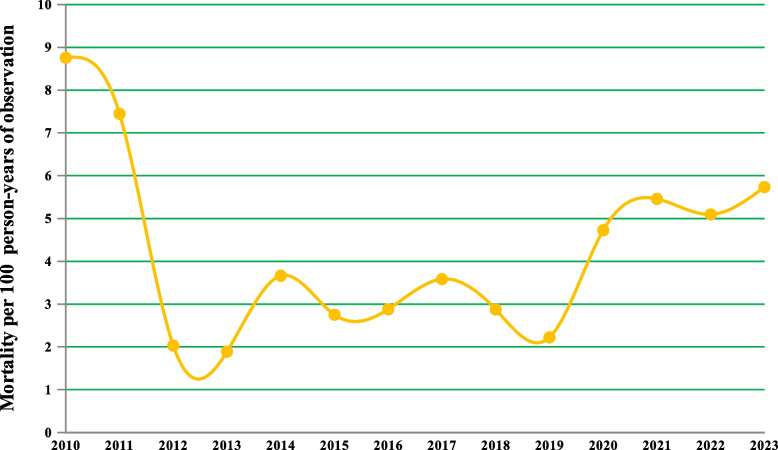
Trends in estimated death rates per 100 person-years of observation among adult patients initiating highly active antiretroviral therapy in Ethiopia, 2010 to 2023

As shown in Fig. 7, an overall significant decline in mortality from 8.76 deaths per 100 PYO (in 2010) to 5.73 deaths per 100 PYO (in 2023) was observed.

#### Predictors of mortality among patients initiating highly active antiretroviral therapy

Table 4 summarizes the pooled hazard ratio of 13 variables (these can be categorized into demographic-related, clinical-related, laboratory-related, and behavioral-related variables) which were found to be predictors of survival among patients receiving HAART in Ethiopia. These were, patients aged ≥ 45 years (HR, 1.70 [95% UI,1.10 to 2.63]), being female (HR, 0.82 [95% UI, 0.70 to 0.96]), history of substance use (HR, 3.10 [95% UI, 1.31 to 7.32]), HIV positive status non disclosure (HR, 3.10 [95% UI,1.31 to 7.32]), cluster of differentiation 4 + T- count < 200 cells/mm3 (HR, 3.23 [95% UI, [2.29 to 4.75]), anemia (HR, 2.63 [95% UI, 1.32 to 5.22]), World Health Organisation classified HIV clinical stages III and IV (HR, 3.02 [95% UI, 2.29 to 3.99]), undernutrition (HR, 2.24 [95% UI, 1.61 to 3.12]), opportunistic infections (HR, 1.89 [95% UI, 1.23 to 2.91]), tuberculosis coinfection (HR, 3.34 [95% UI, 2.33 to 4.81]),bedridden or ambulatory (HR,3.30 [95% UI, 2.29 to 4.75]), poor adherence (HR, 3.37 [95% UI,1.83 to 6.22]), and antiretroviral drug toxicity (HR, 2.60 [95% UI, 1.82 to 3.71]).

**Table 4:** Analysis of predictors of mortality among HIV-infected adult patients after initiation of HAART in Ethiopia

**SN**	**Predictors**	**Comparison**	**No. of studies**	**HR (95% UI)**	***P*.value**	**Heterogeneity**		**Egger’s test**
						**I**^**2**^** (%)**	***P*.value**	***P*.value**
1	Age (in years) (*n* = 2446) [39–42, 51, 59, 65, 66]	≥ 45 vs. < 45	8	1.70 (1.10, 2.63)	0.017*	85.6	< 0.001	0.376
2	Sex (*n* = 5581) [36, 37, 41, 42, 48, 59, 61, 63, 66–68]	Female vs. male	11	0.82 (0.70, 0.96)	0.015*	17.4	0.278	0.147
3	Residence (*n* = 7445) [36, 37, 63, 65, 66, 68, 69]	Rural vs. urban	8	1.2 ( 0.77, 2.03)	0.373	89.1	< 0.001	0.982
4	Substance use (*n* = 5030) [39, 59, 63, 67, 70]	Yes vs.no	5	1.62 (1.00, 2.63)	0.051*	74.4	0.004	0.014
5	HIV-positive status disclosure (*n* = 6366) [39, 41, 51, 54, 59, 66]	Novs. yes	6	3.10 (1.31, 7.32)	0.010*	94.3	< 0.001	0.068
6	CD4 + T-cell count (*n* = 8315) [24, 30, 37, 40–44, 47, 48, 51, 55, 59, 61, 63, 66]	< 200 vs. ≥ 200 cells/mm^3^	16	3.23 (2.29, 4.75)	< 0.001*	88.8	< 0.001	0.260
7	Hemoglobin (in gram/dl) (*n* = 2745) [37, 39, 41–44, 47, 48, 51, 53, 54, 59]	< 10 vs. ≥ 10	12	2.63 (1.32, 5.22)	0.006*	94.9	< 0.001	0.692
8	HIV clinical stages (*n* = 5581) [23, 30, 36, 37, 40–43, 47, 48, 51, 53, 55, 58, 59, 61, 65–70]	III and IV vs. I and II	22	3.02 (2.29, 3.99)	< 0.001*	85.6	< 0.001	0.009
9	Undernutrition (*n* = 5587) [30, 41, 43, 47, 48, 51, 53, 54, 58, 59, 66]	Yes vs.no	11	2.24 (1.61, 3.12)	< 0.001*	82.1	< 0.001	0.172
10	Opportunistic infections (*n* = 7445) [23, 24, 36, 37, 40–42, 51, 54, 55, 59, 63, 66, 69, 70]	Yes vs.no	15	1.89 (1.23, 2.91)	< 0.004*	90.5	< 0.001	0.551
11	Tuberculosis co-infection (*n* = 4884) [23, 39, 41, 43, 48, 51, 55, 58, 68, 70]	Present vs. absent	10	3.34 (2.33, 4.81)	< 0.001*	79.8	< 0.001	0.076
12	Functional status (*n* = 8315) [24, 30, 37, 40–44, 47, 48, 51, 55, 59, 61, 63, 66]	Bedridden or ambulatory vs. working	16	3.30 (2.29, 4.75)	< 0.001*	88.3	< 0.001	0.26
13	Ever took CPT (*n* = 3044) [24, 40, 42, 43, 54, 58, 59, 66, 69]	No vs. yes	9	1.68 (0.94, 3.02)	0.082	94.9	< 0.001	0.926
14	Ever took TPT (*n* = 1546) [23, 41, 47, 48, 58, 66]	No vs. yes	6	1.79 (0.69, 4.63)	0.231	94.6	< 0.001	0.837
15	Adherence to HAART (*n* = 5569) [24, 39, 41–44, 47, 51, 54, 55, 59, 69]	Poor/fair vs. good	12	3.37 (1.83, 6.22)	< 0.001*	95.9	< 0.001	0.592
16	Antiretroviral drugs toxicity/adverse effects (*n* = 5587) [42, 43, 47]	Yes vs.no	3	2.60 (1.82, 3.71)	< 0.001*	0.0	0.577	0.679

*Abbreviations*: *UI* uncertenity interval, *CPT* Cotrimoxazole preventive therapy, *HR* hazard ratios, *HIV* human immune deficiency virus, *TPT* tuberculosis preventive therapy, *TB* tuberculosis, *WHO* World Health Organization

Interpretation: *indicates a statistically significant variable at *P* ≤ 0.05

## Discussion

The findings of this systematic review and meta-analysis revealed that 4,050 deaths were registered among adult patients initiating HAART in Ethiopia, corresponding to a cumulative incidence of 10.13% and pooled mortality incidence density of 4.25 per 100 PYO. Our finding was by far higher than the rate of mortality reported from India (3.12 deaths per 100 PYO) [71] and a multiregional study in Africa and Asia (2.7 deaths per 100 PYO) [72]. However, the current pooled estimate was lower than the individual study estimates from studies conducted in rural settings of South Africa (7.5 deaths per 100 PYO) [73], India (8.1 deaths per 100 PYO) [74], and Uganda (12 deaths per 100 PYO) [75]. The duration of follow-up of the cohorts, year of publication, geographic and cultural barriers, study sizes, and level of engagement in the implementation of HIV programs might partly explain the discrepancy in survival.

Moreover, subgroup analysis revealed a steady decrease in the mortality incidence rate (from 4.15 deaths per 100 PYO before the universal test and treatment strategy to 4.50 deaths per 100 PYO after) following the full-scale implementation of the WHO’s universal test and treatment policy in the country. This is congruent with the strategic approach implemented in Uganda, where a baseline six-month mortality rate of 3.3% decreased by 1.6% after the universal test and treatment policy [76] and a threefold decrease in mortality in Cameroon [77]

Regarding prognostic factors, age, and sex were the two important demographic predictors of mortality identified in this review. First, increased age (45 years or older) was correlated with increased hazards (70% higher) of mortality compared with individuals aged < 45 years. The current findings are congruent with those of studies conducted in other countries [78, 79]. This may be because immunosenescence leads to increased susceptibility to infections in the elderly population and decreased ability to eradicate OIs [80], which in turn decreases the survival of persons receiving HAART. This study also revealed that female patients had an 18% lower mortality risk than male patients. This is in line with a study in Cameroon, where men had twice the risk of mortality compared to females [81], a systematic review by Gupta et *al*. [82], which found that men were more likely to die early in the course of treatment, and a study in rural settings of Uganda, found that females had a 45% lower risk of mortality [83]. Nevertheless, research reports by Woldegeorgis et *al.* [84], and Nicastri et *al*. [85] found little evidence for sex differences, which requires a powerful study. Although an in-depth examination is needed, these differences could be attributed to disparities in socioeconomic status, healthcare-seeking behaviors, treatment adherence, biological differences, and risky sexual behaviors.

Patients who had reported a history of substance use while taking HAART had a 62% higher risk of mortality compared to patients who did not. Research findings consistent the with current findings were reported in Vietnam [86], in which substance use increased the risk of non-AIDS deaths among patients on HAART. Another study indicated that a history of smoking (twice), and alcohol use (25 to 35% higher) were correlated with a decreased life expectancy despite effective HAART [87]. This is because substance consumption, particularly alcohol consumption, interferes with HAART adherence and adverse drug reactions, culminating in decreased HAART effectiveness and, therefore, the survival of patients on treatment.

HIV-positive status disclosure significantly and positively affects HAART adherence through social support and self-efficacy, which in turn contributes to increased quality-adjusted life years of PLHIVs on HAART [88–90]. A systematic review by Yehualashet et *al*. [91] indicated that the pooled national estimate of HIV-positive status disclosure among adult PLHIVs was lower in Ethiopia than in developing countries. This study found that PLHIVs with non-disclosure status had threefold higher mortality hazards than those who disclosed their status. This finding is consistent with that of a study conducted in China [89].

Decreased immunity was another factor related to the increased risk of mortality identified in this study. In line with studies conducted elsewhere [10, 12, 17, 92–95], the mortality hazard was 3.23 times higher among patients whose baseline CD4 + T-cell count was < 200 cells/mm3 than among patients whose baseline CD4 + T-cell count was greater. Although symptoms of HIV can appear at any time during HIV infection, the spectrum of the more severe and life-threatening complications of HIV infection, such as disseminated tuberculosis, which is indicative of a severe defect in cell-mediated immunity in poor settings, occurs as the CD4 + T-cell count declines, more importantly in patients with CD4 + T-cell counts < 200/μL, which decreases the survival of patients receiving HAART.

Anemia was another predictor of mortality in this systematic review. Patients whose baseline hemoglobin level was < 10 g per deciliter had 2.63 times higher hazards of mortality compared to patients who had a hemoglobin level of ≥ 10 g per deciliter In line with our findings, a study in HIV-infected patients from across Europe indicated that anemia is a strong independent marker of clinical prognosis [96]. The results supporting our findings were also reported in Senegal [12], Zambia [8], and Tanzania [94]. Anemia is common throughout HIV infection, and the causes are likely to be multifactorial and may be the direct result of HIV infection harboring underlying opportunistic neoplasms such as lymphoma, OIs such as systemic fungal, and mycobacterium infections, bleeding (gastrointestinal malignancy/severe infection), and poor dietary intake (vitamins such as cobalamin and folate, iron, and general nutritional deficiencies). In addition, antiretroviral and other medication toxicities are associated with bone marrow suppression, further challenging the survival of HIV-infected patients receiving HAART. According to a systematic review by Negesse et *al*. [97] three of ten HIV-infected adult patients on HAART had anemia in Ethiopia, which underscores anemia is a formidable challenge in HIV - infected Ethiopian patients.

Patients with advanced HIV clinical stages (III/IV) at presentation to chronic HIV care and treatment are three times more likely to die than those with mild or asymptomatic HIV clinical stages (I/II). Findings from studies conducted in a rural center in the Far-North province of Cameroon [81], Uganda [98], four sub-Saharan African countries (Côte d'Ivoire, Malawi, South Africa, and Zimbabwe) [99], and Jamaica, analysis of national surveillance data [100] revealed increased hazards of mortality in these patients compared with the general population. This is because life-threatening OIs and malignancies occur at the advanced WHO HIV clinical stage and remain the major drivers of HIV-related mortality and morbidity in PLHIVs.

Undernutrition was another predictor of mortality in this study. Undernourished patients (BMI < 18.5 kg per m2) had 2.24 times higher hazards of mortality compared to patients whose baseline BMI was 18.5 kg per m2 or greater. The effects of HIV on nutrition have been well studied, and a recent meta-analysis revealed that the prevalence of undernutrition among adults initiating HAART was 23.74% in SSA, with the highest (60%) and lowest (8.3%) burdens in Ethiopia and Kenya, respectively [101]. Findings in agreement with our study have been reported elsewhere in Africa [8, 13, 14, 94] and Haiti [102]. The possible justification emanates from the synergistic effect of both HIV and undernutrition; HIV causes poor appetite secondary to chronic inflammation, and enteropathy interferes with nutrient absorption from the gastrointestinal tract [103]. Undernutrition, in turn, accelerates the progression of the clinical stages of HIV because of the direct effect of undernutrition on immunity, all of which negatively affect the survival of HIV-infected children [104, 105].

We found 89% higher hazards of mortality among patients who exhibited OIs than among those who did not. Similarly, research supporting the current finding was reported from India, which stated that patients with any OI before the start of HAART were 2.3 times more likely to die in comparison to patients without any OIs [71]. In Ethiopia, ART is initiated for all HIV-infected patients as rapidly as possible, irrespective of their immunological status; up to 50% [106] of patients present for care and treatment at late clinical stages, with acquired immune deficiency syndrome-defining OIs. Furthermore, a recent meta-analysis revealed that the burden of OIs was high with a pooled prevalence of 43.97% among adult patients receiving HAART [107].

The study found that patients with TB co-infection at the start of HAART were three times more likely to die compared to those without TB at baseline [71]. Moreover, another study found that TB significantly predicts early mortality in adults on HAART in low and middle-income countries [82]. The increase in plasma HIV ribonucleic acid levels during active TB, a leading cause of death worldwide in HIV infection, maybe a possible explanation.

HIV-infected adult patients whose baseline functional status was bedridden or ambulatory had three times higher hazards of mortality compared to patients whose functional status was working. In agreement with our findings, bedridden patients had twice higher hazards of mortality in Kenya [108], threefold in Nepal [109], and in India [74]. This is because late-stage HIV patients have already developed severe forms of OIs and neoplasms, which are responsible for deteriorated quality of life and restricted daily activities, all of which affect their survival despite HAART.

Medication Adherence was another predictor of mortality identified in this review. Patients who had poor or fair adherence to HAART were three times more likely to die compared to patients who had good adherence. Findings in line with our study were reported from India [71, 110], and Canada [111]. A high level of sustained medication adherence is required to suppress viral replication, improve immunological outcomes, decrease OIs, minimize hospitalization and inpatient death, decrease the risk of developing ARV drug resistance, and reduce the risk of transmitting HIV [84, 107, 112].

Last, patients who experienced ARV drug toxicity after initiating HAART had 2.6-fold higher hazards of mortality compared to those who did not. In agreement with the current findings, a systematic review by Mouton et *al*. [113] found a hospital mortality proportion of 2.5% to 16% among HIV-infected adults on HAART in SSA. According to a report from research conducted in seven teaching hospitals in Ethiopia, 22% of HIV-positive patients experience mild to life-threatening ARV drug toxicity [114]. ARV drug toxicity is one of the major causes of non-adherence after initiating HAART, resulting in treatment failure and hospitalization.

## Strengths and limitations of the study

To our knowledge, this systematic review and meta-analysis is the first to estimate the pooled person-time incidence rate of mortality, describe the trends in death rates over time by comparing strategies before and after the universal test and treatment, and investigate potential risk factors associated with death in HIV-infected adults receiving HAART in Ethiopia. Methodologically, the study was adequate as sufficient primary studies were found and a large number of study sizes with fairly sufficient follow-up time were required for outcomes to occur, all of which increased the precision of the study and the true estimate of the mortality rate in adults initiating HAART in Ethiopia. This study had some limitations. First, some statistical heterogeneity was observed; therefore, interpretation of the results in the context is required.

## Conclusion and recommendations

In conclusion, despite almost 20 years of HAART initiation in Ethiopia, the mortality rate remains high. Therefore, patients must be counseled and monitored for enhanced medication adherence, ARV toxicity, and non-AIDS-related predictors of mortality like substance use. Furthermore, screening efforts are essential in the early detection and management of tuberculosis and other OIs, earlier initiation of HAART, and due attention to patients presenting with symptomatic HIV, and anemia, nutritional interventions for undernourished adults, and encouraging partner notification.

## Implications of findings

Our findings have important implications for the provision of comprehensive HIV care and treatment for adults with HIV infection in Ethiopia. Furthermore, with strong conviction, the findings of this meta-analysis contribute to the provision of evidence that can be utilized by researchers, policymakers, clinicians, and other stakeholders in resource-poor settings.

### Supplementary Information

Supplementary Material 1.

Supplementary Material 2.

Supplementary Material 3.

Supplementary Material 4.

## Data Availability

All data supporting the findings of this study are available within the paper and its Supplementary Information.

## Abbreviations

AIDS: Acquired immune deficiency syndrome
ARV: Antiretroviral therapy
CD: Cluster of differentiation
HAART: Highly active antiretroviral therapy
HIV: Human immune deficiency virus
HR: Hazard ratios
OIs: Opportunistic infections
PLHIV: Persons living with the human immune deficiency virus
PYO: Person-years of observations
SNNPR: The Southern Nations Nationalities Peoples region
SSA: The sub-Saharan African countries
TB: Tuberculosis
UI: Uncertainty interval
WHO: The World Health Organization
