# Supplementary material for: Highly active antiretroviral therapy is necessary but not sufficient. A systematic review and meta-analysis of mortality incidence rates and predictors among HIV-infected adults receiving treatment in Ethiopia, a surrogate study for resource-poor settings
Source: BMC Public Health. 2024 Jun 28;24:1735. doi: 10.1186/s12889-024-19268-1 (PMC11214252; doi:10.1186/s12889-024-19268-1)
Supplement: Supplementary file 2 — Supplementary Material 2. [file 12889_2024_19268_MOESM2_ESM.docx]

S1 table : search details

| Search terms | Query |
| --- | --- |
| “ mortality”, “death”, “survival”, “HIV/AIDS”, “Human immune deficiency virus”, “acquired immune deficiency syndrome”, “ART”, “antiretroviral therapy”, “HAART”, “highly active antiretroviral therapy”, “prevalence,” “proportion”, “incidence”, “associated factors”, “predictors”, “determinants”, “adults ”, adolescents,“Ethiopia” | mortality OR death OR survival AND HIV/AIDS OR Human immune deficiency virus OR acquired immune deficiency syndrome AND ART OR antiretroviral therapy OR HAART OR highly active antiretroviral therapy AND prevalence OR proportion OR incidence AND associated factors OR predictors OR determinants AND Ethiopia AND ((english[Filter]) AND (adolescent[Filter] OR all adult[Filter])) |

8190
