# Supplementary material for: Highly active antiretroviral therapy is necessary but not sufficient. A systematic review and meta-analysis of mortality incidence rates and predictors among HIV-infected adults receiving treatment in Ethiopia, a surrogate study for resource-poor settings
Source: BMC Public Health. 2024 Jun 28;24:1735. doi: 10.1186/s12889-024-19268-1 (PMC11214252; doi:10.1186/s12889-024-19268-1)
Supplement: Supplementary file 3 — Supplementary Material 3. [file 12889_2024_19268_MOESM3_ESM.docx]

**S2Table** : Quality assessment of studies using JBI’s critical appraisal tools designed for Cohort studies

| Study | JBI’s critical appraisal questions  Score | | | | | | | | | | | | Score | Overall Appraisal |
| --- | --- | --- | --- | --- | --- | --- | --- | --- | --- | --- | --- | --- | --- | --- |
|  | Q1 | Q2 | Q3 | Q4 | Q5 | | Q6 | Q7 | Q8 | Q9 | Q10 | Q 11 |  |  |
| Gebremichael [30] | Y | Y | Y | Y | | U | Y | Y | Y | Y | Y | Y | 10 | Included |
| Tsegaye and Worku [31] | Y | Y | Y | Y | | Y | Y | Y | U | Y | Y | Y | 10 | Included |
| Hambisa et al [32] | Y | Y | Y | Y | | Y | Y | Y | Y | Y | Y | Y | 11 | Included |
| Abebe et al [33] | Y | Y | Y | Y | | Y | Y | Y | N | Y | Y | Y | 10 | Included |
| Alemu and Sebastián [34] | Y | Y | Y | Y | | Y | Y | Y | Y | Y | Y | Y | 11 | Included |
| Hailemariam et al [35] | Y | Y | Y | Y | | Y | Y | Y | Y | Y | Y | Y | 11 | Included |
| Girum et al [36] | Y | Y | Y | N | | Y | Y | Y | Y | Y | Y | Y | 11 | Included |
| Belay et al [37] | Y | Y | Y | Y | | y | N | Y | Y | Y | Y | Y | 10 | Included |
| Tesfaye et al [38] | Y | Y | Y | Y | | Y | N | Y | N | Y | Y | Y | 10 | Included |
| Kebede et al [39] | Y | Y | Y | Y | | Y | Y | Y | Y | N | N | N | 9 | Included |
| Wondimu et al [40] | Y | Y | Y | Y | | Y | Y | Y | N | Y | Y | Y | 10 | Included |
| Barata et al [41] | Y | Y | Y | Y | | Y | Y | Y | N | Y | Y | Y | 10 | Included |
| Teshale et al [42] | Y | Y | Y | Y | | Y | Y | Y | N | Y | Y | Y | 10 | Included |
| Tesfayohannes et al [43] | Y | Y | Y | Y | | Y | Y | Y | N | Y | Y | Y | 10 | Included |
| Ahunie et al [44] | Y | Y | Y | Y | | Y | Y | Y | N | Y | Y | Y | 10 | Included |
| Mulissa et al [45] | Y | Y | Y | Y | | Y | Y | Y | N | Y | Y | Y | 11 | Included |
| Setegn et al [46] | Y | Y | Y | Y | | Y | Y | Y | N | Y | Y | Y | 10 | Included |
| Birhanu et al [47] | Y | Y | Y | Y | | Y | Y | Y | N | Y | Y | Y | 10 | Included |
| Eticha and Gemeda [48] | Y | Y | Y | Y | | Y | Y | Y | N | Y | Y | Y | 10 | Included |
| Biadgilign et al [49] | Y | Y | Y | Y | | Y | Y | Y | N | Y | Y | Y | 10 | Included |
| Tadesse et al [50] | Y | Y | Y | Y | | Y | Y | Y | N | Y | Y | Y | 10 | Included |
| Workie et al [51] | Y | Y | Y | Y | | Y | Y | Y | N | Y | Y | Y | 10 | Included |
| Fekade et al [52] | Y | Y | Y | Y | | U | Y | Y | Y | Y | Y | Y | 10 | Included |
| Mengesha et al [53] | Y | Y | Y | Y | | Y | Y | Y | U | Y | Y | Y | 10 | Included |
| Birhanu et al [54] | Y | Y | Y | Y | | Y | Y | Y | Y | Y | Y | Y | 11 | Included |
| Seyoum et al [55] | Y | Y | Y | Y | | Y | Y | Y | N | Y | Y | Y | 10 | Included |
| Salih et al [56] | Y | Y | Y | Y | | Y | Y | Y | Y | Y | Y | Y | 11 | Included |
| Yohannes et al [57] | Y | Y | Y | Y | | Y | Y | Y | Y | Y | Y | Y | 11 | Included |
| Damtew et al [58] | Y | Y | Y | N | | Y | Y | Y | Y | Y | Y | Y | 11 | Included |
| Nigussie et al [59] | Y | Y | Y | Y | | y | N | Y | Y | Y | Y | Y | 10 | Included |
| Getaneh et al [60] | Y | Y | Y | Y | | Y | N | Y | N | Y | Y | Y | 10 | Included |
| Digaffe et al [61] | Y | Y | Y | Y | | Y | Y | Y | Y | N | N | N | 9 | Included |
| Abuto et al [62] | Y | Y | Y | Y | | Y | Y | Y | N | Y | Y | Y | 10 | Included |
| Tachbele and Ameni [63] | Y | Y | Y | Y | | Y | Y | Y | N | Y | Y | Y | 10 | Included |
| Sapa et al [64] | Y | Y | Y | Y | | Y | Y | Y | N | Y | Y | Y | 10 | Included |

Y –Yes;N-No;U -Unclear-Question. Overall score is calculated by counting the number of Y’s in each row. Q1)Were the two groups similar and recruited from the same population?Q2) Were the exposures measured similarly to assign people to both exposed and unexposed groups? Q3) Was the exposure measured validly and reliably? Q4) Were confounding factors identified? Q5) Were strategies to deal with confounding factors stated? Q 6) Were the groups/participants free of the outcome at the start of the study (or at the moment of exposure)? Q7)Were the outcomes measured validly and reliably? Q8)Was the follow-up time reported sufficient to be long enough for outcomes to occur? Q9)Was the follow-up complete, and if not, were the reasons for the loss to follow-up described and explored? Q10)Were strategies to address incomplete follow-up utilized?, Q11) Was appropriate statistical analysis used?
